# From Influenza Virus to Novel Corona Virus (SARS-CoV-2)–The Contribution of Obesity

**DOI:** 10.3389/fendo.2020.556962

**Published:** 2020-10-06

**Authors:** Indranil Bhattacharya, Chafik Ghayor, Ana Pérez Dominguez, Franz E. Weber

**Affiliations:** ^1^Oral Biotechnology and Bioengineering, Department of Cranio-Maxillofacial and Oral Surgery, Center for Dental Medicine, University of Zurich, Zurich, Switzerland; ^2^Centre for Applied Biotechnology and Molecular Medicine, University of Zurich, Zurich, Switzerland; ^3^Zurich Centre for Integrative Human Physiology, University of Zurich, Zurich, Switzerland

**Keywords:** obesity, SARS-CoV-2, adiposity, COVID-19, novel corona virus, adipocytes

## Abstract

From the beginning of 2020, the governments and the health systems around the world are tackling infections and fatalities caused by the novel severe acute respiratory syndrome coronavirus (SARS-CoV-2) resulting in the coronavirus disease 2019 (COVID-19). This virus pandemic has turned more complicated as individuals with co-morbidities like diabetes, cardiovascular conditions and obesity are at a high risk of acquiring infection and suffering from a more severe course of disease. Prolonged viral infection and obesity are independently known to lower the immune response and a combination can thus result in a “cytokine storm” and a substantial weakening of the immune system. With the rise in obesity cases globally, the chances that obese individuals will acquire infection and need hospitalization are heightened. In this review, we discuss why obesity, a low-grade chronic inflammation, contributes toward the increased severity in COVID-19 patients. We suggest that increased inflammation, activation of renin-angiotensin-aldosterone system, elevated adipokines and higher ectopic fat may be the factors contributing to the disease severity, in particular deteriorating the cardiovascular and lung function, in obese individuals. We look at the many lessons learnt from the 2009 H1N1 influenza A pandemic and relate it to the very little but fast incoming information that is available from the SARS-CoV-2 infected individuals with overweight and obesity.

## Introduction

In the last 50 years, obesity has gradually shaped into a pandemic. Obesity (body mass index (BMI), ≥30 kg /m^2^) is a combination of genetic, behavioral and environmental variables, and the number of affected individuals has doubled in more than 70 countries. It is not just restricted to developed nations; it is also present in low to middle-income countries (1). With 100 million obese children and 670 million obese adults worldwide, the challenges of tackling obesity involving health, social and economic issues are paramount, and no country is able to reverse the obesity epidemic so far (2, 3).

Many obese individuals may not show any adverse health condition at the onset but if not controlled, checked or reversed, obesity could contribute and develop other co-morbidities such as cardiovascular diseases, diabetes and cancers (3, 4). Many respiratory issues like obstructive sleep apnea, asthma and chronic obstructive pulmonary disease (COPD) have been correlated with obesity (5). Moreover, the incidences of acute respiratory distress syndrome (ARDS) is increased in parallel with body mass (6). Besides causing non-communicable conditions, in recent years it is suggested that excess adiposity is an ideal set-up for acquiring and spreading of communicable diseases, in particular viral infections (7). Moreover, obesity is also linked to urinary tract, periodontitis, nosocomial and surgical infections (8).

The hallmarks of obesity include increase in hypertrophy and hyperplasia of adipocytes, ectopic fat deposition and adipose inflammation (9, 10). Besides adipocytes and preadipocytes, the other cell types present in adipose tissue comprise of endothelial and resident immune cells (11). During the progression of obesity, the dynamics and the functionality of the adipocytes change leading to altered levels of secreted adipokines and a rise in pro-inflammatory immune cells (12).

Undernutrition, which is still prevalent in many developing countries, was suspected to be the responsible factor for the spread of infection (13). However, observations during the H1N1 influenza virus pandemic indicated that overnutrition along with sedentary lifestyle (factors that mediate obesity) increase the risk of infection. A study by the World Health Organization (WHO), with H1N1 infection involving 70,000 individuals from 19 countries provide evidence that obesity, particularly morbid obesity (BMI > 40), is a risk factor for severe disease (14). Emerging strong evidence suggests that excess adiposity and chronic inflammation increase the susceptibility to infection (15). With elevated levels of pro-inflammatory cytokines, the immune system is dysregulated making it difficult to combat infection (8). A correlation between obesity and infections is suggested by humans and rodent studies (16, 17) and reflects the reduced ability of immune cells from obese individuals to fight viral infection. However, the molecular mechanisms for this correlation are elusive and might even vary between viral infections.

Obesity is an expensive condition costing the society and the health system billions of dollars in treatment (18). With the increased frequency of viral infections, the cost of obesity gets even higher. At this time, when the world is facing a pandemic with the novel corona virus (SARS-CoV-2), it is important to use our current understanding on the connection between viral infection and obesity. In the light of the lessons, we learned from the H1N1 influenza A virus pandemic in an obese setting, this review discusses why obese individuals are at risk during this SARS-CoV-2 pandemic.

## Obesity and Immune System

In healthy adipose tissue, adipocytes and the resident leukocytes maintain a balanced homeostatic state and a steady communication either through messenger cytokines or through cell-cell contact (19). Both the innate and adaptive immune cells reside in the adipose tissue to maintain an anti-inflammatory environment and obesity disturbs this situation (20, 21). Indeed, in obese adipose tissue an increase in macrophage accumulation along with a rise in TNF-α and IL-6 inflammatory molecules has been reported (22, 23). Obesity also results in an increase in ectopic fat accumulation in the bone marrow, which is the site where immune cells develop (24). Thus, the interplay of adipocytes and immune cells is altered in obesity compromising immune cell function and giving rise to inflammation.

Not just resident immune cells, the circulating cells are also affected by obesity. In this regard, the total leukocyte and monocyte count in the blood was shown to be increased in obese individuals as compared to lean counterparts (25). Moreover, the circulating peripheral blood mononuclear cells (PBMCs) secrete higher levels of TNF-α and lower levels of the anti-inflammatory IL-10 in obese individuals establishing a permanent “low-grade inflammatory state” (26). Toll like receptors (TLRs) play a crucial role in innate immune system and their activation in PBMCs from obese individuals indicate an impaired ability to express anti-viral type 1 interferons (IFNs), namely IFN-α and IFN-β (27). The circulating PBMCs differentiate into tissue-resident macrophages, which represent a large proportion of immune cell population in the adipose tissue. It is suggested that with an increase in hypertrophied adipocytes and rise in adipose tissue inflammation, the macrophages switch to a pro-inflammatory M1 type (28). Other immune cells, such as those that mediate the adaptive immune response are also affected by obesity (29). The rise in T cell subpopulations such as Th1 and Th17 cause a pro-inflammatory response in obese adipose tissue (30–32). The pro-inflammatory state in obesity is further enhanced by the depletion of regulatory T cell (Tregs), which is associated with infiltration of immune cells and a rise in inflammation (33). Thus, in obesity the proportion of pro-inflammatory immune cells are increased and together with inflammation from hypertrophied adipocytes they create a robust localized and systemic inflammation.

Adipose tissue was traditionally considered a long-term energy storage organ, but it is now appreciated that it orchestrates metabolic functions by the secretion of adipokines such as adiponectin and leptin (34). Moreover, adipokines have immunomodulatory roles and obesity disturbs this function (35–37). Plasma levels of leptin are highly correlated with BMI in both rodents and humans (38, 39) and influence T cell proliferation and Th1/ Th17-dependent cytokine secretion (40). Leptin affects fat and glucose metabolism and is linked to elevated free fatty acids and glucose levels in obesity and diabetes (38). Such high levels of glucose suppress the anti-viral type 1 IFN production in PBMCs (41, 42) and increase the reactive oxygen species in T cells (43). Elevated free fatty acids activate TLRs, induce inflammatory cytokines in circulating monocytes and enhance inflammation in T cells (44–47).

Thus, excess adiposity mediated by the changes in the levels of cytokines, adipokines and metabolites derails the immune response and shifts the balance to a pro-inflammatory state, which most likely favors and promotes infection.

## Influenza Virus Infection in Obesity

Globally 250,000 to 500,000 individuals die of influenza virus and 3 to 5 million individuals, both children and adults, are severely affected annually by this highly contagious virus (48). Cold-weather months and low relative humidity due to room heating promotes the spread of this virus, which is known to cause severe respiratory tract infection along with rapid onset of high fever, cough, and possible headache, sore throat, body aches, nausea and/ or vomiting (49).

The genetic material of this virus is segmented RNA, which is surrounded by a host lipid envelop and is decorated on the surface by hemagglutinin and neuraminidase viral peptides, which help in attachment to the host cell (50). Influenza A, B, C, and D are the four strains, and A and B are the common strains that infect humans. Most human cases are caused by H1N1 and H3N2 influenza A virus strains (51) and to escape the host immunity, the virus goes through antigenic shift and drift causing seasonal influenza epidemics (50).

One hundred years back, the influenza virus triggered the 1918 “Spanish Flu” pandemic, which caused an extraordinary mortality of 50–100 million deaths (52). However, in those days obesity was not wide spread in the society, rather undernutrition was an issue. Since then other pandemics have taken place, which were caused by zoonotic transfer of the virus from animals to humans, but none of those created such an adverse impact as the Spanish Flu in 1918.

After the 2009 pandemic of influenza A (H1N1) virus, an in-depth analysis of the data revealed that obesity was an independent risk factor for increased morbidity and mortality (53). Obesity is often associated with reduced lung volume, abnormalities in respiratory muscle function and gas exchange compounded by sleep apnea and chronic inflammation (5, 54), and this restricted lung function seems highly conducive for the influenza virus infection and thereby injury in the lungs. Recently, increase in BMI positively correlated with adiposity in airway wall, wall thickness and inflammation causing asthma-related death (55). Influenza virus pathogenicity is not just restricted to lungs (56); it also worsens the cardiovascular function (57).

Using rodent models of obesity, many studies have been performed with influenza virus to understand why obesity is a risk factor for infection. High-fat diet-induced obese (DIO) mice infected with influenza virus develop greater lung damage and inflammation, and exhibit a higher mortality rate (58). Increased leptin levels in DIO mice caused severe lung injury and anti-leptin antibody improved the survival of H1N1 infected obese mice (59). The genetic models of obesity such as the leptin deficient ob/ob mice and leptin receptor-deficient obese db/db mice have increased susceptibility to H1N1 virus infection (60). Increased leptin levels in obesity may promote infection by lowering anti-viral type 1 IFN through the activation of suppressor of cytokine signaling-3 (SOCS-3) expression (42, 61). Indeed disruption of SOCS-3 expression provides protection against influenza A virus infection (62).

The role of antiviral IFNs has also been investigated as it is the key to immunity, and suppressed IFN production could be the link for increased infection in obesity (42). In mice, the reduced IFN response in obese condition was shown to create an ideal microenvironment permitting diversity and emergence of virulent strains and treatment with recombinant IFN reduced the viral diversity (63). Most of the studies on the effect of obesity during infection investigated the lungs, which is the primary site for influenza virus infection. The natural killer (NK) cells assist in eliminating infected cells, and infection in obese mice had diminished NK cell cytotoxicity, lower expression of IFN α/β and delayed expression of pro-inflammatory cytokines like IL-6 and TNFα in the lungs (17), indicating lower immune response. DIO mice had reduced influenza specific CD8+ memory T cells post-infection along with a reduction in leptin receptors in the lungs, suggesting possibility of lung injury (64). Obesity also affected the presence of monocytes, lymphocytes and antigen presentation by dendritic cells during infection leading to impairment of immune response in the lungs (65). However, in contrast to the lungs, adipose tissue have a higher infiltration of inflammatory cells, and increased levels of TNFα, MCP-1, and IL-6 (66), which are known to cause complications like insulin resistant and also dysfunctional adipose tissue. Besides inflammatory mediators, metabolic profiling of serum, adipose tissue, liver, lungs, urine and feces from obese mice infected with influenza virus showed differential increase in specific metabolites such as certain lipids, ascorbate, glucose, 3-hydroxybutyrate, all known to affect the T cell population (67), thus lowering the adaptive immune response.

In humans, the extent of adiposity determines the severity of disease post-infection. The influenza viral RNA was detected in aerosol and there was a positive association with viral aerosol load (indicating shedding) and BMI (68). Higher BMI was shown to be an added risk factor for hospitalization and during the influenza months of the year, the incidences of hospitalization with obese individuals increased (69, 70). Moreover, obese individuals had a longer hospital stay. Indeed, the data suggests that obesity slows the recovery since it delays the clearance of the influenza virus load and prolongs the shedding duration causing long-term transmission (71). Obese individuals infected with H1N1 virus had a two-fold higher chance to end up in Intensive Care Unit (ICU) (72). A multicenter study with 144 ICUs in Spain revealed that obesity was associated with higher ICU resource consumption and hospitalization in H1N1 infected individuals (73). Among patients admitted to ICU due to H1N1 infection, obese and morbidly obese patients were more likely to develop pneumonitis compared to non-obese patients (74). With underlying co-morbidities like obesity and diabetes even younger patients over the age of 20 years had higher hospitalization and mortality (53, 75, 76). Thus, the influenza pandemic data shows that obesity is a risk regardless of age and possible other co-morbidities such as hypertension and diabetes could enhance the severity of the disease.

## Influenza Virus Vaccination in Obesity

Vaccination is still the best way to prevent the risk of developing infections. Obesity, however, interferes with the protection by vaccination against infectious diseases (7) and indeed obese individuals were reported to show a greater decline in influenza-specific antibody titers at 1 year after vaccination (77). Moreover, vaccinated obese adults had twice the risk of developing influenza despite a good antibody titer in response to the vaccine (78). PBMCs challenged *ex vivo* with vaccine virus strain showed that obese individuals had decreased CD8^+^ T cell numbers along with a decline in influenza antibody titers and lower protection to vaccination (77). In a further study by the same group, PBMCs from overweight and obese individuals when stimulated *ex vivo* with H1N1 virus showed a defect in CD4^+^ and CD8^+^ T cell activation despite intact dendritic cell functions, suggesting that both overweight and obesity negatively impact the immune function (79).

The above studies with influenza virus show that excess body fat elevates inflammation, weakens the immune response particularly in the lungs, hampers vaccination and creates an ideal environment for influenza viral infection and spreading.

## Is Obesity a Risk Factor for SARS-CoV-2?

Coronaviruses (CoVs) are a family of enveloped RNA viruses that infect mammals and birds. The last two decades saw the emergence of novel coronaviruses that triggered human fatalities. Firstly, in 2002/2003 there was the outbreak of SARS-CoV and then in 2012, the World saw an outbreak of Middle East respiratory syndrome coronavirus (MERS-CoV). Both these viruses originated from animals and infected humans (80). Though these viruses did not spread efficiently from human to human, both SARS and MERS had a high fatality rate of 9.5 and 34.4%, respectively (81). Comorbidities were reported to exist in MERS infected individuals and in one such study obesity was associated with 17% of the MERS infected hospitalized patients (82). The 2002 SARS-CoV infection lowered anti-viral IFNα/β, upregulated TNF receptor, IL-8 and hypoxia related genes (83). Obesity also lowers the IFNs activity and increases hypoxia in adipocytes leading to a heightened inflammation (42, 84). Hence, the combination of the viral infection and obesity probably creates an ideal platform that favors a pro-viral inflammatory “cytokine storm” by lowering the anti-viral immune response.

The 2019 novel coronavirus, referred to as SARS-CoV-2, is closer to SARS as it also binds to human angiotensin-converting enzyme 2 (ACE2). ACE2, which is also referred to as ACE2 receptor acts as the entry point for the coronavirus to infect a wide range of human cells. The viral spike protein S present as transmembrane protein in the viral envelope has a strong affinity for human ACE2 receptor. ACE2 is expressed in a wide variety of human tissues with different expression levels including the small intestine, testis, kidneys, heart, thyroid, lungs, brain and the adipose tissue (85–88). ACE2 is known to generate vasodilator angiotensin-(1-7) from vasoconstrictor angiotensin II, and expression of ACE2 in adipocytes is known to protect against obesity-mediated hypertension (89). ACE2 induces an anti-obesity effect by stimulating brown adipocytes and through browning of white adipose tissue (90). Moreover, activator of ACE2 is reported to reduce adiposity (91). Thus, it is highly probable that the beneficial effects of adipose ACE2 are lost after the binding with SARS-CoV-2. Loss and/ or reduction in ACE2 activity means increase in Ang II levels and indeed Covid-19 patients show increased levels of plasma Ang-II, cardiovascular complications and a linear association with lung injury (92).

Recent studies suggest that COVID-19 patients with obesity are at a greater risk for hospitalization. Obesity was suggested as an independent risk factor for SARS-CoV-2 infection and the proportion of patients in France who required invasive mechanical ventilation increased with BMI (93). In Spain, obesity was the strongest comorbidity among patients admitted to ICU (94). In a retrospective analysis of COVID-19 patients admitted to a hospital in Wuhan, China, 88% of non-survivor patients had a BMI >25 kg/m^2^ (95). A report from Intensive Care National Audit and Research Center (ICNARC, UK) from 19 June, 2020 show that of the 9,272 COVID-19 patients admitted in critical care units 39.3% were obese & 35% were overweight (https://www.icnarc.org/Our-Audit/Audits/Cmp/Reports). In USA, which is witnessing an explosion of SARS-CoV-2 infection, 40% of the adult population is obese. In an Editors Speak Out column Ryan et al., suggest that, in USA, obese individuals are at risk of infection and obesity could be an independent risk factor for COVID-19 (96). Data of 5,700 COVID-19 patients from the New York City area with median age of 63 years show that many patients had comorbidities such as obesity (41.7%), hypertension (56.6%) and diabetes (33.8%) (97). A letter communicated by Lighter J et al., on the COVID-19 patient data from a New York City hospital report that in patients younger than 60 years, obesity is a risk factor for hospital admission (98). The authors indicate that of the 3,615 patients admitted in the hospital 38% were with a BMI of ≥30 kg/m^2^ and that obesity increases the likelihood of admission in critical care unit by 2 times as compared to those with lower BMI in the same age group. Similar data was reported in young obese individuals with H1N1 infection (53). With all the data pouring in, it is still unclear why SARS-CoV-2 infection deteriorates the health and increases the hospitalization of COVID-19 overweight and obese patients.

The driving features of SARS-CoV-2 are reduced levels of anti-viral IFNs along with high levels of chemokine and cytokines like IL-6 (99). Even though inflammation of the lung is the primary symptom in COVID-19 patients, the expression of ACE2 in lungs is very moderate (88). Even with moderate ACE2 expression, it may be enough to drive the lung inflammation. Li et al., found no difference in the expression levels of ACE2 in the lungs of healthy individuals and those with chronic respiratory disease, suggesting that in both groups SARS-CoV-2 could infect the lungs (100). Hence, the additional underlying conditions such as obesity could contribute to worsen the lung function. Obesity is related with chronic obstructive pulmonary disease (COPD) and ACE2 expression is significantly increased in COPD than non-COPD subjects (101). Besides causing acute respiratory distress syndrome (ARDS) and pneumonia, SARS-CoV-2 also causes cardiovascular complications (102).

Until now, most of the studies on SARS-CoV-2 have focused on the lungs even though other organs such as adipose tissue and heart have higher expression of ACE2 (88). Whether a cross talk exists, by which infection in adipose tissue affects the lung and heart function is a matter of investigation. Based on the present findings, we suggest the following possible factors through which excess adiposity in the presence of SARS-CoV-2 deteriorate health, in particular the lungs and cardiovascular function.

### Inflammation

Expression of inflammatory molecules (IL-6, TNFα) and C-reactive protein are increased in overweight and obesity (103, 104) and increased inflammation is shown to deteriorate the lung and the cardiovascular function. High levels of IL-6 is associated with lung lesions after SARS infection (105) and increased levels of circulating IL-6 is connected with systolic blood pressure in hypertensive subjects by increasing the expression of angiotensinogen and angiotensin II receptor (106). Data from COVID-19 patients show that a cytokine storm exist in severe patients and highly elevated IL-6 levels in the serum contributes to the cytokine release syndrome (107, 108), which could probably reduce the immune response in lungs by inhibiting the ability of dendritic cells to activate T cells (109).

### Renin-Angiotensin-Aldosterone System (RAAS)

The ACE2-Ang II balance is altered by both obesity and by SARS-CoV-2 infection. Activation of RAAS results in higher blood pressure and elevated levels of Ang II induces endothelial dysfunction (110). Moreover, SARS-CoV-2 viral elements have been reported within endothelial cells along with an accumulation of inflammatory cells (111), which could further worsen the cardiovascular system. In the lungs, downregulation of ACE2 receptors is shown to cause immune-cell infiltration and expression of inflammatory cytokines leading to lung edema and acute lung failure, which appear to be mediated by Ang II (112).

### Adipokines

Leptin levels are increased in obesity and elevated leptin levels deteriorate lung and cardiovascular function (59, 113). It is reported that COVID-19 patients with high BMI have significantly higher levels of serum leptin (doi: https://doi.org/10.1101/2020.04.30.20086108).

### Ectopic Fat

An increase in ectopic fat, in obesity, may worsen the function of the organ that is in close proximity to the adipose tissue. For example: the epicardial adipose tissue (EAT) which is in close proximity with the myocardium could be a possible source for myocarditis and indeed myocarditis is reported in COVID-19 patients (114). Fat embolism in lungs through the action of the released lipid droplets from necrotic adipose tissue could be another way by which obesity affects lung function upon viral infection (115).

Even though there are several similarities between influenza and SARS-CoV-2 virus and both these viruses worsen the lung and cardiovascular function in obese setting, some differences also exist. Both viruses utilize different entry points to bind and enter the cells. Even though there is a report which suggests that influenza virus utilizes ACE2 to induce acute lung damage (116) however for SARS-CoV-2 infection, ACE2 is the main route to infection. It is highly important for clinicians and health workers to distinguish between the two types of infections, which have almost the same readouts. It is suggested that COVID-19 patients have a higher median age, higher proportions male subjects, a history of cardiovascular diseases as compared to H1N1 patients (117). The case fatality risk (CFR) suggest that COVID-19 is more severe than H1N1 infected hospitalized individuals (118).

Even though the information coming out on SARS-CoV-2 is very rapid, some open questions remains.

#### Some Important Open Questions

1. Does SARS-CoV-2 infect the adipose tissue and enhance the inflammation?
2. Do COVID-19 obese individuals have a longer virus shedding duration and therefore need longer isolation period?
3. If SARS-CoV-2 reduces memory CD8^+^ T cells in obesity, then how effective a vaccine will be for obese individuals?
4. Will countries with less obese individuals per million of population, have a lower morbidity and mortality rate to SARS-CoV-2?

## Conclusion

**Figure 1:**
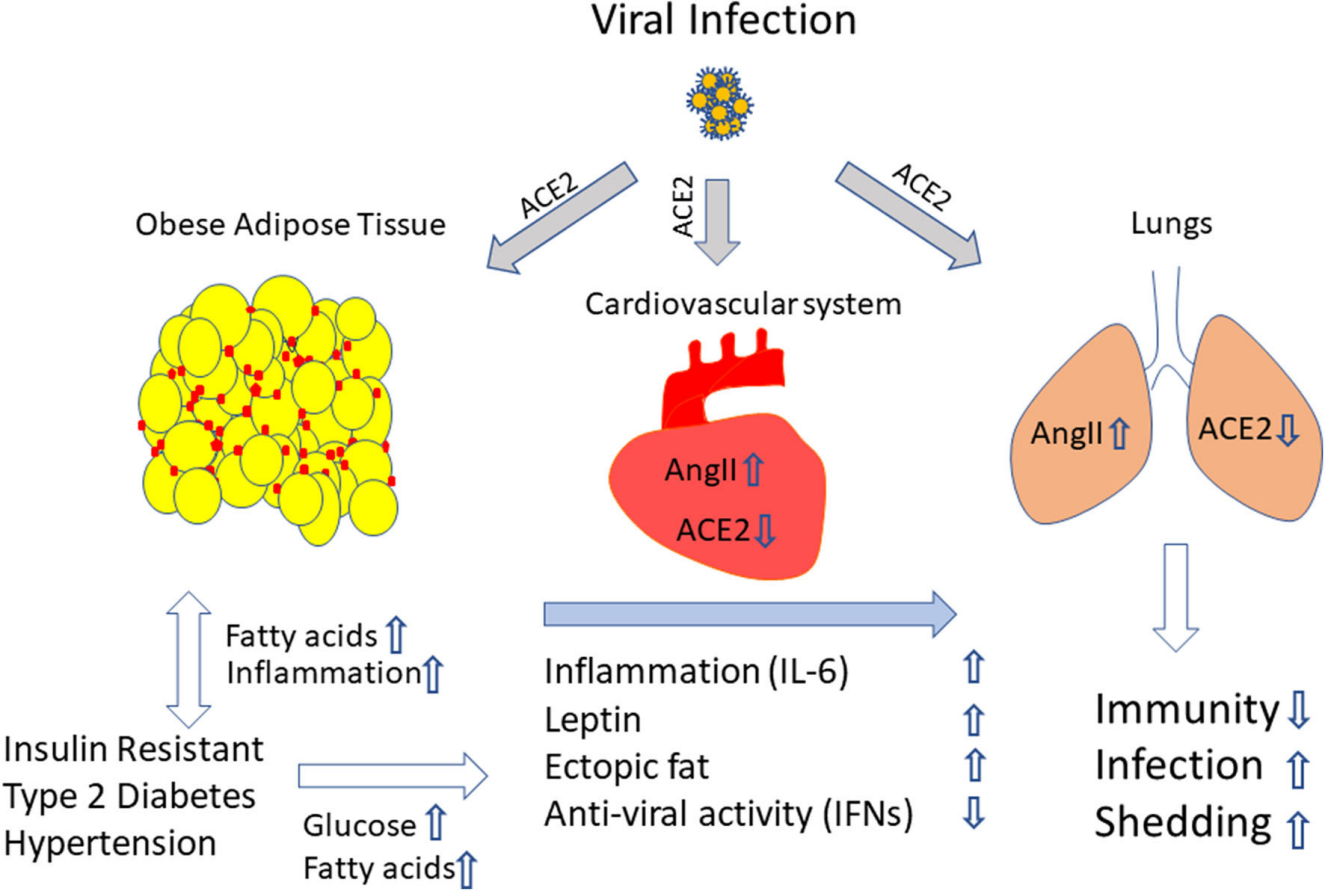
Excess adiposity provides an ideal setting to promote viral infection. We propose that obesity and its associated conditions elevate the cytokines, adipokines and RAAS and increase ectopic fat accumulation. When infection sets in, in this case with SARS-COV-2, through ACE2 receptor on the adipose tissue, the lungs, the heart and the blood vessels, the virus enters, mediates a cytokine storm and creates an imbalance in AngII/ACE-2 levels, elevates leptin levels and lowers anti-viral molecules like IFNs. Moreover, adipose tissue a might serve as a reservoir for viral persistence, which could be the possible source of continuous viral shedding and systemic inflammation. The combination of all the factors deteriorates lungs and the cardiovascular system, weakens the immune response, and promotes viral shedding. Yellow circle indicate adipocytes, red marks indicate inflammatory cells, upwards and downwards arrows indicates upregulation or downregulation of molecules and/ or effect.

Based on the studies, we suggest that obesity favors viral infection (both influenza and COVID-19). At this stage of the COVID-19 pandemic, it is evident that obesity and infection together build up a cytokine storm that determines the severity of the disease. Moreover, increased inflammation in particular elevated IL-6 levels, activation of RAAS, rise in Ang II levels, higher leptin and increased ectopic fat favor COVID-19 disease progression and severity, and thus worsen the lung and cardiovascular function (Figure 1). The lower levels of anti-viral IFNs and weakened immune response in COVID-19 patients make the fight against this infection more difficult and the effect of a potential vaccine questionable in obese individual. Therefore, overweight and obese individuals should be informed that they belong to a risk group and should avoid the risk of SARS-CoV-2 infection by any means.

## Author Contributions

IB, CG, AP, and FW gave inputs and helped in drafting the manuscript.

## Conflict of Interest

The authors declare that the research was conducted in the absence of any commercial or financial relationships that could be construed as a potential conflict of interest.
